# Vocational Rehabilitation: Supporting Ill or Disabled Individuals in (to) Work: A UK Perspective

**DOI:** 10.3390/healthcare4030046

**Published:** 2016-07-16

**Authors:** Andrew Frank

**Affiliations:** 1 Vocational Rehabilitation Association, 42 The Croft, High Barnet, Herts EN5 2TL, UK; andrew.frank1@btinternet.com; Tel.: +44-0-1895-268-847; +44-077-1420-3928; 2 Department of Clinical Sciences, College of Health and Life Sciences, Brunel University London, Middlesex, UB8 3PH, UK

**Keywords:** vocational rehabilitation, return to work, disability, work, Employers

## Abstract

Work is important for one’s self-esteem, social standing and ability to participate in the community as well as for the material advantages it brings to individuals and their families. The evidence suggests that the benefits of employment outweigh the risks of work and are greater than the risks of long-term unemployment or sickness absence. Individuals may be born with physical or intellectual disadvantages (e.g., cerebral palsy), or they may be acquired during childhood or adult life. Some progressive conditions may present in childhood or adolescence (e.g., some muscular dystrophies) and these need to be distinguished from those presenting later in life (e.g., trauma, stroke). Vocational rehabilitation (VR) thus takes three forms: preparing those with a disability, health or mental health condition for the world of work, job retention for those in work and assisting those out of work into new work. Important components of VR consist of the attributes of the individual, the skills/knowledge of their health professionals, the knowledge and attitudes of actual or potential employers and the assistance that is provided by the state or other insurance facility. Charities are playing an increasing role.

## 1. Introduction

Vocational rehabilitation (VR) is an enormous topic. Its principles are used in a wide variety of ways in virtually all medical situations where the worlds of work and health/ability coincide. This review cannot cover all the variety of VR situations, nor the outcomes of VR interventions, which would demand a systematic review of thousands of references, which is beyond the scope of this review. It aims, however, to review broadly the meaning and applications of VR in common clinical situations and to facilitate understanding of the role that VR practitioners can play in improving the provision of rehabilitative health care for those whose physical or mental impairments impede their working lives.

The skills of VR practitioners can assist those whose working ability is affected by congenital impairments, those who acquire ill health acutely and need assistance in returning to work (RTW), and for those with long-standing conditions. It thus covers individuals with a disability, health or mental health condition.

Whilst the evidence base for VR is limited in the United Kingdom (UK) literature, its value has been clearly established using varying methodologies [1,2,3]. As is often noted in rehabilitation research, packages of care are often evaluated without the individual ingredients being evaluated separately. This review will try and accentuate those ingredients of VR that are most often used in clinical practice.

## 2. Is Work Important?

For many years, the clear advantages of work (e.g., payment) may have been masked by the obvious disadvantages (sometimes long hours for low pay with risks to physical and mental health). This has been clearly enunciated by John Ruskin who wrote “Which of us, in brief words, is to do the hard and dirty work for the rest—and for what pay? Who is to do the pleasant and clean work, and for what pay? Who is to do no work, and for what pay?” [4]. It is arguable that, in the last half of the twentieth century, the welfare state, as it developed in the UK, provided a cushion for all potential workers that tended to accentuate the advantages of receiving benefits rather than seeking the benefits of paid employment. In any event, the UK government commissioned an independent review of the scientific evidence into whether work is good for health and well-being [5]. In this review, the same terminology will be used as by Waddell and Burton—the term “work” will imply the application over time of effort, skills and knowledge put into either paid or unpaid activities [5]; and “employment” usually takes the form of a contractual relationship for financial (and/or other) remuneration [5].

Waddell and Burton found extensive evidence to support the beneficial effects of work in terms of adequate economic resources to facilitate full participation in society, meeting important psychosocial needs and being central to individual identity, social roles and social status. Thus, employment is the main driver of social gradients in physical and mental health and mortality [5]. Conversely, there is a strong association between worklessness and poor health in terms of higher mortality, poorer general health and poorer mental health [5]. Dame Carol Black summarised the advantages of employment: “For most people, their work is a key determinant of self-worth, family, esteem, identity and standing within the community, besides, of course, material progress and a means of social participation and fulfillment” [6].

If this is true for the general population, it is likely to be just as true for those disadvantaged in the workforce due to physical or mental impairments. It follows that caring societies should be giving every assistance to these individuals to become work ready, remain in employment or find new employment if they become unemployed, as has been argued in the UK [7,8].

## 3. What Is Rehabilitation?

Rehabilitation has multiple meanings and definitions, but is used in this review primarily in the context of ill health or long-term impairment of body or mind. Throughout this document, the concepts of the World Health Organisation International Classification of Function [9] will be used. This views the consequences of ill health/injuries in terms of residual “impairments”, which may then influence the ability of the individual to function at individual, employment and/or societal levels. The ability to function may be influenced again by personal or environmental factors, one of which will be the working environment.

**Figure 1:**
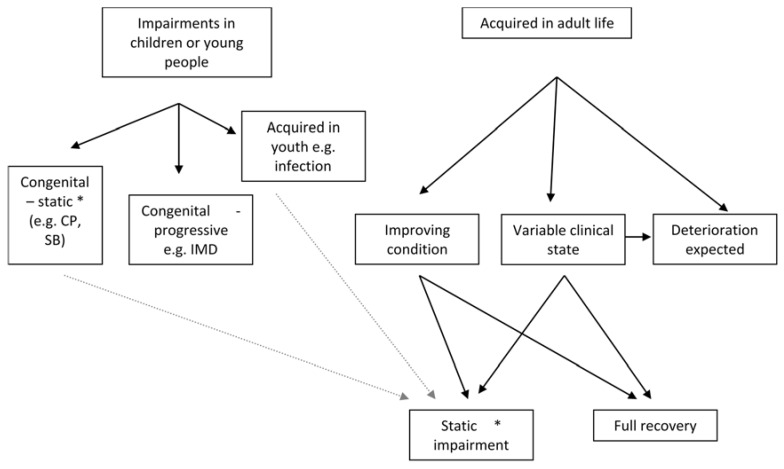
Potential clients for VR (vocational rehabilitation) services. CP, cerebral palsy; SB, Spina Bifida; IMD, Inherited metabolic disorders; *, May deteriorate slowly over time.

Rehabilitation in its broadest sense defies definition as it embraces a philosophy—essentially one that states that there are many ways of supporting those with physical or emotional impairments. How the philosophy is put into practice will vary in relation to the underlying impairments—see Figure 1. The principles followed within a rehabilitation context would include a goal-setting process with agreed goals between the rehabilitation professional(s) and the individual being assisted.

**Figure 2:**
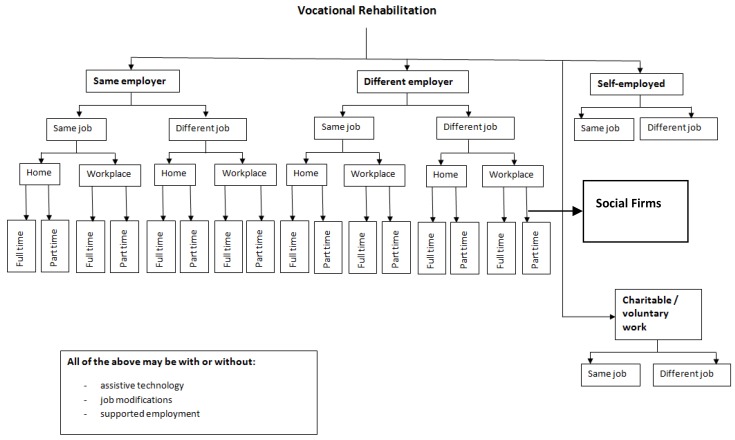
Suggested routes to help individuals back into work.

The initial discussion about an individual’s work needs to take place at the beginning of any acute illness. It may be limited to identification of the nature of any employment, advice to remain in contact with their employer and, when the illness is severe, reassurance that there are many ways to support workers back into employment when the clinical state allows. Health professionals must avoid making ill-informed statements about the likelihood of a return to work (RTW). This is not just a theoretical possibility—it is the experience of many with disabilities [10]. Early VR is important, as individuals (and their professional advisers) tend to only see themselves through the eyes of their current job. They do not think—as VR professionals think—about changes to their employment—see Figure 2.

## 4. What Is Vocational Rehabilitation (VR)?

VR is that part of the rehabilitation process that relates to employment (or other useful occupation for those unable to earn an income). It embraces vocational goals as part of the rehabilitation plan [11]. Waddell et al have described VR as “whatever helps someone with a health problem to stay at, return to and remain in work: it is an idea and an approach as much as an intervention or a service” [3]. This emphasises the rehabilitation philosophy discussed above. However, a more technical professional definition is of a “*a process*, which *enables persons* with functional, psychological, developmental, cognitive and emotional impairments or health conditions to overcome barriers to *accessing, maintaining or returning to employment or other useful occupation”* [12] (the italicised words are those in common with a shorter British Society of Rehabilitation Medicine (BSRM) definition [13]). More recently, there has been concern that the RTW process is often not sustained [14] and that disadvantaged individuals have difficulty in developing a career pathway after their RTW [14].

The core ingredients of any VR or RTW process are the relationships between the client, the actual or potential employer, the health professional(s) involved and the insurer [15,16,17].

There are three aspects of VR:

- Preparing disadvantaged young people for the world of employment
- Job retention—supporting and maintaining those currently in employment
- Facilitating new work for disadvantaged individuals currently out of employment and unemployed or on ill-health benefits

### Brief Historical Perspective

Historically, rehabilitation was seen as a sequel to medical interventions, the “post-medical rehabilitation treatment” referred to by Beveridge [18]. Rehabilitation services have been promoted after the two world wars and, more recently, the fighting in the Middle East and Afghanistan. However, the concept of rehabilitation after the conclusion of medical treatment is now thought to be outdated and often harmful. Rehabilitation is currently seen to begin at the commencement of an episode of health care and not as a “bolt-on” after completion of medical interventions [13,19,20,21]. It is crucial that rehabilitation is commenced as early as possible in collaboration with the treating clinicians (where different).

Up to the 1980s, facilitating a RTW after illness was seen as a priority for health professionals. For example, patients referred to my back pain clinics during the 1980s were automatically given urgent appointments if they were out of work because of their pain. However, this practice was gradually withdrawn as it was seen to be favouring one section of the patient population against another.

At the same time, it is likely that supporting disadvantaged workers was given less priority, as there were approximately three million unemployed workers during parts of the 1980s, and priority may have been given to support these healthy individuals back to employment. Another practice that developed was increasingly to take disadvantaged individuals out of the workforce by redundancy on medical grounds, knowing that state benefits, e.g., incapacity benefits, would provide financial support (although many other factors were likely to have affected early retirement, e.g., the state of the economy and pension funds etc. [22]). Recipients of incapacity benefits rose massively during the 1990s, reaching nearly 2.7 million by 2003 [23]. This process was seen to be increasingly financially unaffordable by the late 1990s, with, for example, rising numbers of individuals with sickness absence (SA) due to back pain rising to over 100 million days in 1993–1994 [13].The National Health Service (NHS) closed down most of the rehabilitation centres which functioned during the 1980s (many stimulated by the Demonstration Centres in Rehabilitation supported by Sir Keith Joseph), which were designed to speed individuals’ recovery [24]. It was argued that closing these centres may have contributed to the rise in the costs of disability benefits [25].

The end result was that the NHS did not support disadvantaged individuals back to employment, which was seen to be the responsibility of the Department of Employment. This disastrous separation of responsibilities between the Department of Health and the Department of Employment resulted in “ignorance within the NHS of the means of re-integrating individuals into employment” [13]; and that the “NHS had largely lost the culture and skills of facilitating employment as a key element of effective health care” [13]. The “sheer numbers of people on incapacity benefits represents an historic failure of healthcare and employment support to address the needs of the working population” [6].This was clearly unsustainable, and was recognised in 2005 with the appointment of Dame Carol Black to coordinate the workings of these two government departments.

The report by the BSRM in 2000 not only commented on the parlous state of rehabilitation services (including VR) in the UK, but also brought together for the first time the various players involved in the management of SA, which included employers, some of which would have occupational health (OH) and primary care (family doctor) services as well as the various roles of health and rehabilitation professionals [13].

The lack of good NHS rehabilitation services also stimulated the development of private rehabilitation services, mostly paid for by the insurance sector which realised that the lack of good rehabilitation in returning accident victims to work was proving expensive. At the same time, two groups of professionals were formed, representing the two main streams of rehabilitation as it was developing in the private sector—the Case Management Society of the UK [26] and the Vocational Rehabilitation Association, which has recently published the second edition of its standards of practice [12]. Both these bodies now have members from the public sector as well as the private sector.

There is currently a growing interest in VR in the UK expressed by government, health professionals and the charitable sector as discussed below. It is notable that there are now calls for work to be considered a health outcome [27,28].

## 5. Preparing Disadvantaged Young People for the World of Work

Helping disadvantaged young people is difficult as there needs to be close working relationships between the local education and social service departments, local and sometimes specialist health services, equipment providers and often the charitable sector. All these bodies have defined budgets giving scope for endless negotiations between them as to who pays for what [29].

Although the level of education achieved and qualifications gained are crucial to achieving an optimal lifestyle [11,30], other factors are also fundamental. Social and personal development is one such factor [11], which may be assisted through exposure to role models in the areas of motherhood, sport and work. The development of self-confidence can be assisted through such means as sport [31], adventure (e.g., scouting) and, crucially, through work experience [11]. This work experience cannot be the individual who just “makes the tea” but should be meaningful [32]. Internships may lead on to apprenticeships. The charitable (voluntary) sector is often helpful in giving specific advice to individuals with specific conditions, e.g., for those with hearing impairments [33]; and broader charities such as Disability Rights UK offer a wide variety of factsheets, e.g., on traineeship, internship and apprenticeships [34,35]. Volunteering is often a good entry point into the world of work [36,37]. Appropriatecareers advice (e.g., from the National Careers Service [35]) from those with in-depth knowledge of disabled people’s capabilities and the careers of the future is essential, both for young people [32,36] and for older individuals changing careers due to illness [38]. Advice is available from the Disability Employment Adviser from the local Jobcentre Plus office (part of the DWP) about potential employment opportunities as well as support for those seeking self-employment [35]. Advice on self-employment is also available from the Prince’s Trust [35].

For those able to go to university, the Disabled Students’ Allowance may make all the difference, e.g., by covering the cost of a dyslexia specialist who visits the home [35]. Support for disadvantaged students may take many forms, including provision of specialist equipment (e.g., a voice-activated computer), sign-language interpreters, extra tutorial help and extra assistance for assignments, field work or exams, etc. [35].

Whilst there has been concern for some years about the services available in the UK for the transition between childrens’ services and those for adults [39,40,41], little attention appears to have been given from the NHS to the preparation of disadvantaged young people for working life [41]. A number of factors seem important. Planning for an adult world of work needs to begin around the time of puberty, or attending secondary education [11]. Childrens’ services need to plan for the young person to become the centre of the clinic consultation, rather than their parents. This can be difficult for some doctors and parents who have known each other since the birth of a child, e.g., with cerebral palsy (CP). Parents must be confronted with the question as to whether they want their child to become a young adult with, as nearly as possible, the same chance of an independent life as their non-disadvantaged peers? For those parents grasping this issue, allowing increased personal independence, either through the use of assistive technology (AT) or through other means, can greatly enhance the life-chances for their child as they develop into a young adult. The process of “letting go” is greatly facilitated by the use of AT [42]. It has been suggested that “decreasing the physical strain on parents may also facilitate the emotional separation that naturally occurs in most able-bodied youngsters as they mature” [43].

### Equipment and Assistive Technology

The provision of equipment to support physically disabled young people, e.g., that of communication equipment (Alternative and Augmentative Communication (AAC)), can transform the lives of even the most severely impaired individuals, including facilitating employment [44]. AAC is often provided by the NHS but is very costly and poorly funded [29,45]. Equipment provided, although personalised, may not move with the disabled individual when they reach adult life, particularly if funded by education services [29].

Powered wheelchairs, although transforming lives of profoundly immobile individuals [31,46,47] and greatly assisting their carers (caregivers) [46,48], may have long waiting times, even with very stringent eligibility criteria.

Although powered wheelchairs can be provided by the Department for Work and Pensions (DWP) (which was previously the Department for Employment and included other functions) to facilitate working, there is no coordination between the Access to Work Scheme (AtW) (see below) and local wheelchair services. Occasionally, the Voucher Scheme has been used when the wheelchair service has advised on an electric-powered indoor/outdoor wheelchair (EPIOC) deemed most suitable for their needs and the AtW Scheme has funded additional components to facilitate work, e.g., a riser function [49].

These issues are raised, not just because they reflect a lack of planning of equipment provision to support the needs of those with physical impairments (and indeed of the UK’s rehabilitation services in general), but because employment opportunities often relate to the degree of social integration and personal independence achieved by the disabled individual [11], and for many these employment opportunities are “in offices and using computers” [11]. Assistance with computing for those with difficulties using standard computers may be provided by AbilityNet—a charity able to support individuals at home, or employers, in making “reasonable adjustments” to their information technology systems [11]. Any training that may be needed to facilitate the use of specialised software may get the costs reimbursed through AtW [35].

All those who find difficulty in using mobile handsets, e.g., to control a television, should be assessed for environmental control units (ECUs). Together with powered wheelchairs, severely disabled youngsters can control their environment, leave home and meet their friends, etc. [43,50]. Mobile phones give parents some degree of reassurance that they can remain in contact to be available if needed.

## 6. Job Retention (JR)—Issues Present Prior to an Episode of Ill Health

### 6.1. Employers Management Roles

The key facet to job retention lies with the employer [51] and the important ingredients are shown in Table 1.The importance of a sympathetic employer is crucial [17,36,38,52,53,54,55,56,57,58,59,60,61]. Sound management is an essential prerequisite for efficient support for disadvantaged workers [17,57], including a sound health and safety policy [62]. The importance of the work environment has been well understood for many years [13]. One cannot underestimate the role of good management in having sound absence policies which recognise the need for good communication between the employer and the employee [61]. Such policies should facilitate clear differentiation between absence that relates to ill health, other social factors and those which point to the need for disciplinary action. These policies have to be clearly understood throughout the whole organisation. “*Prevention of ill health at work and amelioration of the effects of health on work,* e.g., *through rehabilitation, are essentially management issues”* [63].

### 6.2. Occupational Health (OH)

The term “disability management” is infrequently used in the UK. Its functions are usually the remit of the human resources departments with advice from OH, where available. In the UK, OH is about the health and well-being of the workforce:

- Minimising the adverse effects of work on health
- Mitigating the effects of ill health on work [64].

Where present, OH is provided by a team which may comprise a number of differing health professionals as well as Health & Safety officers, occupational hygienists and ergonomists [28]. Larger services are usually run by consultant physicians in OH. However, OH services in the UK are impoverished, with only 14% of workers in the UK receiving OH support and about 12% having access to OH physicians [28]. Whilst most large employers will provide OH services, it is well recognised that employees in small- and medium-sized companies often have inadequate OH support [65]. If the UK wishes to reduce the 140 million working days lost to SA each year [7], then investment in OH should help reduce unnecessary SA through advice to management as to how to minimise accidents and mental ill health, have appropriate absence monitoring mechanisms and policies, assist in RTW for those off sick and provide early support for workers needing, e.g., physiotherapy or counselling [28].

However, new roles for OH are being defined, with an anticipated greater collaboration between OH and primary care (family doctor) services. The recent formation of the Council for Work and Health is exploring how greater access to the working population can be provided, through many different mechanisms, some of which lie outside traditional OH pathways.

### 6.3. Supervisors and Co-Workers

Polices that are not implemented at shop floor level are useless. The experience of disabled employees to develop an open and supportive culture can benefit all staff [35]. Supervisors clearly are important [1,17,36,66]. If managers/supervisors do not understand and use their absence policy, then inappropriate disciplinary actions or SA may ensue [51,67] and the important role that co-workers can play in the VR process may not be forthcoming [51]. The perceptions relating to the employees belief about the potential for RTW are important and include perceptions relating to their co-workers [33,68,69]. Some organisations involve co-workers in the workplace RTW programme e.g., as “buddies” to assist the employee’s RTW [35,62]. Buddies may provide general assistance when needed to disadvantaged colleagues, or they may have some specific training, e.g., to support a colleague with mild dementia [70]. Supportive co-workers are important for job sustainability [71]. The importance of co-workers is crucial in the RTW process in a wide variety of situations [1,56,72,73,74,75,76,77] and is included in questionnaires assessing relationships between disadvantaged workers and the workplace, often couched in terms of social support, or support from colleagues at work etc. [78,79,80]. Indeed, lack of support from co-workers may be a difficulty in successfully accommodating those with mental health problems [52,81] and other conditions [54] at work and contributes to an increased risk of low back pain becoming long-standing [80]. Conversely, individuals with profound problems can be assisted in their work by supportive co-workers [82,83], even though co-workers themselves may need training and support [84].

### 6.4. Work Instability

There is increasing interest in determining risk factors for work disability, which is of value for employers in reducing SA [17]. Rehabilitation professionals are now recognising “work instability”, “*a state in which the consequences of a mismatch between an individual’s functional abilities and the demands of his or her job”* [85]. Scales have been devised for rheumatoid arthritis [85] and ankylosing spondylitis [86]. Similar scales have been devised to screen office workers [87] and nurses [88] for work instability. These scales can be used also to measure change after interventions. The same group from Leeds University established the need for a vocational practitioner as part of the rheumatology team to facilitate suitable workplace accommodations for their patients with rheumatological complaints [89]. This concept, however, appears valid for many conditions which may fluctuate over time, e.g., mental health issues.

## 7. Job Retention (JR)—Post Prolonged Sickness Absence

The ingredients of JR for those with more complex problems are already well established. There are four important ingredients to successful RTW, consisting of the attributes of the individual concerned, the employer, the health professionals involved and the health insurer [11,15,16]. The nature of the state involvement may vary from country to country, but in the UK relates to the many interventions available from the DWP (summarised by the BSRM [11], but up-to-date information is always best obtained via the DWP website).

After the initial stages of early VR (see above), the approach often used in any preliminary assessment is to assess the difficulties (sometimes referred to as obstacles or barriers) in RTW. In the UK, a “flag” system has been developed, elaborating the original “red flags” which were symptoms and signs found with individuals with low back pain, which suggested that the cause of the pain may need investigation [90]. Other flags have been devised over time to signify non-impairment obstacles for a RTW which may reflect psychological or social factors. There is no significance to the different colours—but together, they summarise the bio-psycho-social model [90] which is now considered the hallmark of good health/rehabilitation practice [91] (see Table 2).

Assuming that there is an effective absence policy, which maintains good contact between employee and employer [61], then the first step in the RTW process is to establish the RTW plan [1,51,60,93], which may be a simple process. Where, however, SA is likely to be prolonged, the RTW plan will consider which of the steps in Table 3 may be helpful in establishing an early RTW. A plan for the first week may also be helpful and may include what the employee does or does not wish to discuss with colleagues [35]. The next step is to establish if there are any components of the employee’s work that (s)he can still perform and, if so, build on that. The advantage to the employer is that long-term SA with its associated costs is reduced if the employer can participate in the employee’s rehabilitation in this way. Whilst the recognition is relatively new that it is best for all concerned to facilitate an early RTW, in spite of an incomplete recovery, the processes that facilitate it are well understood, some being enshrined in law (Equality Act [94]), which insists on reasonable accommodations or job modifications being made [36]. Examples of workplace adjustments include “doing things another way”, e.g., allowing individuals with social anxiety to have their own desk rather than hot-desking, improving ventilation for those with, e.g., heat intolerance, designated parking spaces for those with limited mobility, etc. [35]. Task modification may be crucial, e.g., reducing work pressure and offering further support when pressure cannot be avoided [35]. Financial support may be available to employers through AtW and the support of Jobcentre Plus [35]. The RTW may be organised simultaneously with the provision of physical support (e.g., a graded exercise programme) or counselling and employers should understand the importance of facilitating this [66]. The RTW plan is thus likely to embrace the processes listed in Table 3. Where support from an OH team is available, then that team may well be the key to facilitating the RTW plan [95]. Job satisfaction is a good predictor of an early RTW [96].

One of the key roles of the health professionals involved in the RTW plan is to provide information about prognosis. A good example relates to planning the RTW following a hip or knee replacement. It has been shown that those undergoing such surgery report inconsistent advice regarding expected periods of SA and some had none at all, either preoperatively or postoperatively [95]. A minimum period of 12 weeks seems to have been assumed, irrespective of the presence or absence of physicality in the nature of their employment [95].

Some employees may need further training to support returning to their previous or modified work, or occasionally, for redeployment in another role. Employees can ask for face-to-face training, e.g. on boosting confidence [35].

In some situations, working from home may be helpful either temporarily, as part of the rehabilitation into work [35,66], or permanently. Home working may make sense for many in terms of reducing long and sometimes painful journeys into work and may be in any combination of days at work and days at home. For a minority of individuals, home working may be permanent, particularly if personal independence is problematic and time-consuming, and there are family or other individuals able and willing to support home working. A disadvantage, however, may be that of social isolation. Where good family or other support networks exist, self-employment from home may be a good way forward.

### Government Roles

The government has a key role to play in vocational rehabilitation in providing “top-down” policies that support those providing the services—“bottom-up” provision [23]. The example of legislation has already been discussed (e.g., Equality Act), but the provision of the “Fit note” in replacement of the “Sick Note” has been important [61,66]. It has defined for all practitioners the principle of rehabilitation *that it is not what one cannot do that matters, but what one can do!* There is evidence that the Fit Note has been valued by employers [61]. It allows the general practitioner (family doctor) to suggest options to the employer “a phased RTW, altered hours, amended duties and/or workplace adaptations” [66].

The government provides support for workers who need help in RTW, such as assessing literacy and numeracy skills [37], preparing a CV, preparing for job interviews, funding a suit for an interview, etc. through a variety of schemes. Such support may also be available through the charitable sector [35]. The roles of the Work Programme and Work Choice are outside the scope of this review. Access to Work (AtW) [97] is a highly successful government scheme designed to support disabled individuals and their employers with the adjustments needed for them to start or undertake their work [98]. AtW also, through JobcentrePlus, pays a grant towards extra employment costs resulting from a disability. It has been successfully used for a wide variety of disadvantaged workers. Examples include providing hearing aid-compatible telephones, loop systems and deaf awareness training for staff [33], taking a taxi to work if, e.g. fatigue from multiple sclerosis prevents the individual from travelling to work using public transport when they are otherwise able to work (with or without job modifications (accommodations) [99], paying for a support worker, thus allowing the applicant to use the services of a helper, e.g. for care needs after traumatic brain injury (TBI) [98] and, more recently, addressing workplace stress and mental health problems [100].

In view of the relative lack of OH advice for employers, the government has set up a website providing free health and work advice for employers and telephone helpline to assist with the prevention of SA [101]. For those whose employees are expected to have four weeks of SA, a free referral for OH advice can be obtained from the Fit for Work Service from the DWP [35,102].

## 8. Finding New Work

People seeking to return to a previous occupation following an extended period of leave should usually be offered an assessment of vocational skills by a suitably qualified practitioner. Success is dependent not only on the client’s previous level of education, but also the client’s previous work experience [60]. If those supporting the disadvantaged individual to RTW are not part of a rehabilitation programme, then there should be close liaison with any health professionals involved, e.g., a general practitioner to ensure that all the medical obstacles to RTW are clearly understood and strategies to overcome them adopted. This is particularly likely to be important in certain conditions such as cognitive impairments [11].

For those whose loss of job is related to health-related conditions, some will require rehabilitation from a multiprofessional team and this has been shown to be particularly helpful in Scotland for those with musculoskeletal conditions [103]. Such rehabilitation approaches are likely to be needed for a significant proportion of those currently on incapacity benefits who are found to be potentially able to work.

A vocational assessment —with many similar characteristics as the employment assessment practiced by the DWP [11]—may be highly technical and beyond the scope of this review, but will embrace an individual’s previous education, qualifications, employment, hobbies that might convert into a job and transferable skills; as well as medical/rehabilitation history, social and family circumstances and current state of the labour market, etc. [11]. One of the key factors in determining future job roles is that of the personal inclinations of the potential employee [104], which will not only influence job uptake but also the likelihood of sustaining the new job. Whilst understanding the nature of any new job, the rehabilitation team should be assessing the potential for working at home—see above. Such work may be within employment, or self-employment, and the DWP has a number of support services for those seeking self-employment [35]. Advice about self-employment from those who have achieved it in spite of severe communication impairments has been given [44], quoted by the BSRM [11], and includes the importance of taking practice jobs (work trials), networking with future co-workers and employers, demonstrating competence and learning social interaction skills [11,44].

Other innovative approaches involve Work Integration Social Enterprises, which are social enterprises that focus on helping people with disadvantages into employment; whether that is through job creation, job placement, work preparation, Intermediate Labour Market (ILM) schemes or vocational training.

It is clear from the above that many potential employees will need (re)training to facilitate any RTW. This may be provided directly via the DWP or through an ILM scheme. Such schemes aim to reduce economic inactivity by offering contracts to organisations providing personal development to people facing barriers to employment through a period of supported employment and training. Such schemes are often provided through the charitable sector [105].

In addition to the strategies listed above and in Table 3 for those returning to their previous employment (with or without job modifications), additional components may be needed to assist in preparation for and support in pursuing alternative occupation—See Table 4.

Many people who are assisted back into old or new jobs will require on-going support to ensure the job is secured for the long term. Whilst this is now well recognised, facilitating advancement in a career when an individual is disadvantaged is an area needing further research and assessment.

In reality, many ingredients of successful VR are common to each of the three aspects mentioned above. Some key ingredients are worth highlighting.

## 9. Some Key Ingredients to Successful VR

### 9.1. Client Profile

The importance of the level of education (and qualifications gained), social and personal development and self-confidence have been discussed above. Potential employers’ value skills, e.g., adaptability, communication skills, ability to work collaboratively and decision making, etc. [35]. A reference from an employer gained from, for example, school-based work experience, can be very helpful. Older individuals will have an employment record, which is invaluable. Often, older workers have acquired transferable skills either through the workplace or through hobbies, and such experience may open the doors to new employment. Likewise, a reference from previous employers indicating a good work record is of great assistance to the older worker seeking new employment.

In addition to self-confidence [104] and self-awareness/social skills [11,44,60,106], other innate qualities will influence employability. These include assertiveness [107,108], conscientiousness [109] and motivation [106,109,110]. Resilience, or the ability to bounce back, is another important quality, [35] and VR professionals can enhance this to assist their clients in coping with the disappointments inherent in finding work [108]. Other personality traits may be important, e.g., emotional vulnerability, which may be general or the result of specific events or illnesses [98,104,111]. Locus of control may be important with internal believers having greater RTW rates [112,113,114]. An individual’s illness beliefs can also be fundamental to their attitudes to a RTW [115], and this may be particularly important in areas where there is a high immigrant population [14,104].Thus, the belief in illness having more severe consequences is significantly associated with expectations regarding an RTW [116]. The effects of ethnicity on RTW may be significant, although the reasons may not be clear [117].

These strengths and weakness may be exaggerated by the emotional responses to the client’s situation, which may affect his/her mood. Those in long-term unemployment often experience a depressed mood, which can be improved with good VR programmes [108]. These mood changes may be dramatic, e.g., the presence of severe depression and post-traumatic stress disorder following trauma—treatable diagnoses that are often missed within the primary healthcare system in the UK, in my experience [118,119,120]. For others however, post-traumatic growth may reflect positive responses to trauma or illness [35].

### 9.2. Facilitating the RTW Process

Vocational professionals have an important and sometimes critical role to play in the RTW process. These professionals go under a variety of names, e.g., vocational therapists/consultants/case coordinators/counsellors (VTs) [11,56,93,98,121], or sometimes return to work coordinators [89,122]. Their prime functions include matching the abilities and aspiration of the client with the demands of employment [36,60,93,98]. They may arrange work with local employers [60] or with the voluntary sector [37]. This may lead to more formal work trials [11,44,60]. Worksite visits are usually important in facilitating a RTW [56,59,98,99] and may involve training at the worksite [11,60,98,121]. Such visits greatly facilitate the introduction of job modifications (accommodations). Since all these interventions demand the active involvement of the employer, the employer is now considered part of the rehabilitation process [59].

### 9.3. Job Modifications (Accommodations)

Job modifications are one of the commonest forms of VR, with an enormous reported use. Examples include training in the use of aids/equipment, restricting work to easier tasks/duties after traumatic brain injuries (TBIs) [60], use of round tables during meetings to facilitate lip reading [33], performing both task and environmental analysis to break down tasks into manageable steps and reducing the demands of the job for those with multiple sclerosis [99], environmental modifications to facilitate use of powered wheelchairs by those with CP to save energy for the physical demands of the job [11], specific low vision aids and AT to assist visually impaired individuals in their work [37], ergonomic adjustments for those with arthritis [38,66], use of rest breaks to reduce effects of fatigue after TBI [55] or static work postures in those with neck pain [123].

### 9.4. Early Intervention

The importance of early intervention has already been discussed, as the longer the client is off work, the greater the difficulties in RTW [66]. However, it does have two dimensions. Firstly, the appropriate timing of the health intervention [1,124] and secondly, the early focus in RTW planning [1,66]. Often both factors apply [122]. This is clearly seen in management of musculoskeletal disorders, where they remain common [1,66], but also in the management of TBI [60], multiple sclerosis [99], mental health [100] and chronic pain [122], etc. Good management policies that insist on action being taken on day one of SA, with a referral to occupational health on day 10 of SA, have had dramatic effects on reducing SA in Lanarkshire (Scotland) [125].

### 9.5.On-Going Support

VTs should also provide support after the RTW. Even if they are not needed, the reassurance provided that help is available if needed is very valuable [11,56]. Often, however, such support is crucial, as carefully arranged plans fail, e.g., when the client’s work manager of supervisor changes [60], or if there is a change in the mental health of the client—perhaps even precipitated by events in the workplace (see co-workers above). The best follow-up programmes for those with complex injuries, e.g., TBIs, include reports from the client’s supervisor as well as reviewing the client’s experiences at work [60].

### 9.6. Who Provides the VR?

The increased survival of those having had cancer has stimulated thinking about the levels of support needed. Macmillan Cancer Support, together with the Department of Health, have outlined levels of support as:

- Level 1: Information and support provided electronically or through the printed media
- Level2: One-to-one support through telephone hotlines and digital media
- Level 3: Self-management programmes access during or after treatment
- Level 4: Specialist VR services [126].

However, this ignores the fact that VR services are provided both generically and in specialist areas [127]. There is evidence that some VR specialists need to be expert in just one area of health or impairment, e.g., specialist TBI services, where they are likely to be part of a clinical specialist team [60], services for those with epilepsy [11] or services for those with severe mental illnesses receiving, e.g., individual placement and support [128].

There is a strong private rehabilitation sector (reflecting poor NHS rehabilitation facilities). There, the term case manager is often use for professionals fulfilling the VR (and other coordinating) roles. They are particularly valuable where complex navigation between insurance companies and the legal profession is needed.

Where OH departments are present, they can greatly assist the development of sound policies and facilitate good RTW strategies [17,57,62] (see above).

### 9.7. Charitable or Voluntary Sector

Throughout this review, there have been constant references to the voluntary or charitable sector, which plays a massive role in supporting disadvantaged individuals in the UK. This may reflect the inadequate nature of the NHS rehabilitation services. As in other forms of VR, the sector operates both "top-down” and “bottom-up”. Some charities are generic in that they support a wide range of disadvantaged individuals, e.g., charities that support people with disabilities. Others, however, are highly specific, either in terms of supporting those with a particular condition, or a particular impairment, regardless of any diagnosis.

#### 9.7.1. “Top-Down” Services

Most large charities, particularly generic ones, have a major role in lobbying government on behalf of their members. Those that are related to a particular disease/group of diseases also often work closely with professional bodies, and these links can work at both national and local levels. Disability Rights UK represents “disabled people leading change, working for equal participation for all” and lobbies government with these objectives. Such bodies act as advocates for those they represent at national and sometimes international levels. They also finance research and provide subjects for research studies, including employment opportunities for those with individual impairments [33,37,129]. They also provide advice for families and carers, giving invaluable sources of advice/information, both in leaflets and web-based services.

#### 9.7.2. “Bottom-Up” Services

Examples of charities with local branches working with local NHS services include the National Ankylosing Spondylitis Society and the National Osteoporosis Society, who often work with physiotherapy departments for the provision of exercise groups or local hydrotherapy. Providing advice and support (peer support [35,130,131]) may also be crucial for individuals with specific conditions, e.g., Headway (for those with TBIs), the Multiple Sclerosis Society, Stroke Association, etc. These collaborations between professional and voluntary bodies often result in the provision of guidelines [11] or other valuable sources of advice [33]. Some bigger charities have large websites which allow individuals to consider all the different aspects of employment with an individual condition, e.g., Work and Cancer [132]. All these organisations act as advocates for their members when needed.

Other collaborations may result in the provision of a specialised centre for, e.g., those with neuromuscular disorders [133] which can “enhance independence, create a culture of understanding and empathy facilitating mutual support and self-acceptance and offer advice about employment offering a sense of purpose” [133]. Some charities provide rehabilitation and VR, e.g., for those with TBI, where 15 charities are reported to offer some form of VR in the UK [98].

There are many local Disabled People’s Organisations, which offer a variety of supports for disabled individuals, but some may also offer Work Experience [35]. Residential training and support (for example, for those with CP) may be designed and/or run by general [134] or specific charities supporting those with a particular impairment, e.g., charities supporting those with CP [11].

Charities have a major role in funding equipment, e.g., AAC [29], or special wheelchairs [135]. WhizzKidz is an example of a charity whose purpose is to support children and adults up to the age of 25. They not only provide equipment, but also encourage activities like camping to develop social skills and confidence and work placements for young wheelchair users all across the UK, often in partnership with corporate partners [135]. Many charities also offer support in gaining crucial skills to facilitate RTW, e.g., interview skills, writing CVs, etc. [35].

Social Firms are market-led businesses set up to create good quality jobs for people severely disadvantaged in the labour market [11]. They usually are non-profit organisations, any profit being reinvested in the business for the benefit of its employees.

## 10. Conclusions

Vocational rehabilitation embraces a large number of skills which facilitate employment of those with disability, physical or mental ill health. Although individual characteristics are important, close cooperation between the individual, health/rehabilitation professionals and supportive employers offer the best hope of employment. The government plays a key role through legislation, numerous RTW schemes and specific support for clients and employers in meeting the challenges of ill health/disability in the workplace. For those with severe difficulties resulting from ill health or disability, professionals with both health and employment skills are needed to provide individualised RTW services.

The opinions expressed in this review are those of the author and are not those of the Vocational Rehabilitation Association.

## Figures and Tables

**Table 1:** Employer’s influence on job retention.

The milieu of the organisation (well-managed, happy stable workforce, etc.) Presence of an “Absence Policy” appropriate to the needs of the company (assisting differentiation between absence caused by ill health or other causes) This policy is known and understood by the workforce, including shop floor supervisors and managers The policy is implemented, particularly that part which ensures the maintenance of contact between the employer (or representative) and employee following ill health The policy understands that employees may RTW prior to full resolution of the health/disability issues as part of their rehabilitation (or recuperation, if preferred). This process may involve co-workers who need to support this aspect of the policy Job modifications and phased RTW may be needed Nature of any occupational health provision which should contribute to the above.
From: Vocational Rehabilitation Association letter to Lord Freud March 2011

**Table 2:** The flag system of obstacles inreturning to work [92].

Red—	severity of impairment (a)
Yellow—	psychosocial obstacles (b)
Orange—	those with pre-existing psychological impairments (b)
Blue—	perceived obstacles in the workplace—changeable (c)
Black—	unalterable obstacles—e.g., national agreements (c)
Chequered—	social obstacles (c) [64]
a	Biological
b	Psychological
c	Social
a–c	Reflect components of the “bio-psycho-social” model

**Table 3:** Recommendations for Work Adjustments (fromBritish Society of Rehabilitation Medicine [11]).

Flexibility in hours and/or duties, e.g.,:
Changes to working hours or days Time off to attend health-related appointments Provision of additional breaks during the working day Changes to start/finish times to reduce travel during the busiest times Review/adjustment to the overall level of responsibility of a job role Consideration of an alternative job role
Adaptations, equipment and coping strategies, e.g.,
Help with travel (e.g., designated parking space or taxi through AtW) Provision of home working to reduce travel demands Physical adaptations or re-organisation of the working environment (e.g., to allow wheelchair accessibility) Additional equipment, aids and adaptations (e.g., communication aids/software, specialist seating) Advice on specific symptom management (e.g., fatigue management) Advice/support on the use of coping strategies (e.g., for cognitive impairment)
Additional training, supervision and support, e.g.,:
Job coaching/support worker in the workplace Ongoing support from a co-worker A “buddy” trained to respond to specific needs (e.g., seizure) in the workplace Additional training, supervision and/or support (e.g., mentoring, advocacy, etc.) Education for supervisor, manager and colleagues about the condition and its effects Advice/support for supervisor/manager (e.g., to assist work planning/prioritising) Advice/support for supervisor, manager & colleagues Regular reviews with supervisor/manager (e.g., to assist work planning/prioritising) Additional support for colleagues in the workplace Off-site support (e.g., from a rehabilitation service or vocational practitioner)

**Table 4:** Additional components to finding alternative or new work (from [11]).

Graded progression of work-related activities Careers guidance and vocational counselling to identify a suitable job Links with any local Employers’ Partnership or Employers’ Forum “Work taster” to sample alternative avenues of occupation Assisted job selection, search, application, interviews, etc. Voluntary work trials * Permitted work options Supported work placements * * Such trials or work placements require:
Job matching with the skills of the person Needs of the person are communicated clearly to the employer Health & Safety training and insurance cover provided by the employer Provision of on-site job coaching when needed Person is guided and supported in adapting strategies to the workplace Trial/placement monitored closely through contact with the person and the employer Trial/placement does not impact negatively on either the person or their relatives
